# The changing dynamics of ant-tree cholla mutualisms along a desert urbanization gradient

**DOI:** 10.1371/journal.pone.0280130

**Published:** 2023-03-31

**Authors:** Shannon C. Lynch, Amy M. Savage

**Affiliations:** Rutgers University Camden, Camden, NJ, United States of America; Universidade Federal de Uberlandia - Campus Umuarama, BRAZIL

## Abstract

Urbanization, among the most widespread and multifaceted anthropogenic change drivers, exerts strong influences on a diversity of ecological communities worldwide. We have begun to understand how urbanization affects species diversity, yet we still have limited knowledge about the ways that species interactions are altered by urbanization. We have an especially poor understanding of how urbanization influences stress-buffering mutualisms, despite the high levels of multivariate stress that urban organisms must overcome and the importance of these interactions to the fitness of many organisms. In this study, we investigated the effects of urbanization on a mutualism between tree cholla cacti (*Cylindropuntia imbricata)* and visiting ants. We first examined how plant size, ant species composition, and ant activity varied on *C*. *imbricata* across an urbanization gradient (urban, suburban, wild) in and around Albuquerque, NM. Ant species composition and activity varied significantly across the urbanization gradient, with ant communities from wildlands having the highest activity and the most dissimilar species composition compared to both suburban and urban sites. In contrast, plant size remained constant regardless of site type. We then experimentally assessed how nectar levels influenced ant aggressive encounters with proxy prey (*Drosophila melanogaster* larvae) on *C*. *imbricata* across urban and wild sites. Ants were more likely to discover, attack, and remove proxy prey in wild sites compared to urban sites; they also performed these behaviors more quickly in wild sites. Nectar supplementation had weaker effects on ant aggression than urbanization, but consistently increased the speed at which aggressive behaviors occurred. Future studies that examine nectar quality and herbivorous arthropod abundance may help explain why this strong difference in ant composition and aggression was not associated with lower plant fitness proxies (i.e. size traits). Nevertheless, this study provides unique insight into the growing body of work demonstrating that mutualisms vary significantly across urbanization gradients.

## Introduction

Anthropogenic change drivers are causing habitats to become more stressful and less predictable globally [1]. Urbanization is among the most far-reaching and multifaceted of these anthropogenic change drivers; organisms living in cities must simultaneously cope with fragmentation, urban heat islands, changing water dynamics, pollution, and altered nutrient profiles [2, 3], creating environments unique from surrounding wildlands. For example, one study found that microclimate in Phoenix, AZ and Baltimore, MD were more similar than their respective surrounding wildlands [4]. As urbanization continues to spread, a major challenge for conservation is to understand how ongoing urbanization affects individuals, communities [5], and interactions between the species that remain in these environments.

Mutualisms, which are characterized by positive fitness benefits for both interacting partners, with a mix of positive and negative effects on component metrics of fitness [6, 7], have been historically understudied both within and outside of cities [6, 8]. These changes could lead to cascading effects on local communities, ecosystem processes, and surrounding habitats, making this knowledge even more essential [9]. Ant-plant food for defense mutualistic interactions are widespread, with plants from 100 different families participating in these kinds of mutualisms across a diversity of habitats and geographic locales [10]. Therefore, it is imperative that we assess how urban stress influences the dynamics of mutualisms.

The dynamics of mutualisms are likely different in cities compared to nearby undisturbed landscapes, but it is not yet clear whether we should expect organisms to invest greater or fewer resources into particular mutualisms as urban stress increases [11]. Most studies on the influences of urbanization on plant-insect mutualisms have focused on reproductive mutualisms, especially pollination. These studies have found increasing quantity, but reduced quality of pollination services, likely due to the higher abundances of generalists relative to specialists [12] and increased phenological mismatches between plants and their preferred pollinators in cities [13]. While much less studied, stress-ameliorating mutualisms, such as ant-plant interactions, are likely to influence not just the interacting partners, but also the communities in which they are embedded. Moreover, urbanization may have more profound effects on biological communities in deserts by exacerbating present extreme environmental conditions [14]. For example, Mooney et al. (2016) found that *Ligusticum porter* aphids were more abundant in shaded habitats in cities, not because of any direct effects of light on the aphids, but as a result of the influence of light on the aphids’ mutualistic ant guards [15].

In this study, we investigated the dynamics of mutualisms between tree cholla cacti (*Cylindropuntia imbricata* Hawforth 1828) and their ant guards across an urbanizing landscape in and around Albuquerque, NM. Ant-plant food-for-defense mutualisms are considered classic models for facultative mutualisms [11]. In these mutualisms, plants provide extrafloral nectar (EFN) as incentive for ants in exchange for protection against herbivorous arthropods [6, 7]. While exclusion experiments have demonstrated that ants can provide benefits to plants by reducing impacts of herbivory, the magnitude of this benefit has been shown to vary greatly [7, 16]. Moreover, multiple studies have demonstrated that understanding the dynamics of these mutualisms across different levels of nectar availability can facilitate greater understanding of the communities in which they are embedded [8, 15, 17].

The ant-tree cholla mutualism is ideal for our line of inquiry because it occurs in dense patches across both wild and urban systems and because we have extensive knowledge of the dynamics of this mutualism in wildlands. Specifically, Tom E. X. Miller and his colleagues have been documenting demographic patterns of tree cholla plants and dynamics of the tree cholla-ant mutualism for nearly 20 years [18–20]. Many ant-protective mutualisms, including this ant-tree cholla mutualism, involve multiple species. Interactions between these partner species can vary, with outcomes often correlated to partner identity and partner quality [7, 16, 18, 21]. Species who are more aggressive to competitors are more likely to be aggressive to herbivorous arthropods, and thus are superior defenders [21].

To attract more effective ant defenders, plants may produce better quality or quantity of nectar [22, 23]. This response could be more costly to fitness, due to energy and nutrient loss, if a non-defending fauna effectively rob nectar. González-Teuber (2012) found that addition or removal of nectar had pronounced effects on mutualistic interactions. Application of nectar caused increased ant activity and reduced damage from herbivory. Despite this response, it may be a net cost to plants to produce additional nectar if ant protection is weak [24]. Similarly, Savage and Whitney (2011) found that increasing nectar availability led to increased aggression towards prey on EFN-bearing plants from some-but not all-ant species [17]. It remains to be seen how urbanization will influence these behavioral outcomes in the context of this mutualism.

Urbanization exerts unique challenges on a diversity of organisms because cities are stressful across many different environmental axes (e.g. temperature, humidity/precipitation, chemical pollutants, space, etc.). In addition to directly affecting interacting species in mutualisms, urbanization can indirectly alter species interactions by causing changes in the rewards or services that are exchanged in mutualisms. An initial step towards improving our understanding of how organisms cope with multifaceted urban stress is to determine which players are numerically dominant in stressful environments and how dynamics of the mutualism change with urbanization. Once we have these data, we can target experiments to the organisms that are most likely to play a role in changing the dynamics of the mutualism for the interacting partners and influencing the community-wide impacts of the mutualism. Thus, in this study, we first asked: (1) how do plant structural traits and ant composition and activity differ across an urbanization gradient? We predicted that generalist ants would be more common and plants would be smaller as urbanization increased. To address this question, we conducted a survey of ant composition and activity and *C*. *imbricata* size traits across urban, suburban, and wild sites in and around Albuquerque, NM (USA). We then asked: (2) how does ant aggression towards insects on *C*. *imbricata* plants vary across urban and wildland sites? Ant species turnover could lead to less effective defense in urban areas. Alternatively, urban stress could lead plants to invest more heavily in extrafloral nectar rewards, leading ants to provide stronger defense of a more valuable resource. Finally, we asked: (3) how strong are the direct effects of urbanization on this mutualism relative to variation in nectar availability? To address these questions, we performed a nectar supplementation experiment across urban and wild sites and assessed the relative effects of urbanization, extrafloral nectar availability, and their interaction on ant behaviors.

## Materials and methods

### Survey

#### Site selection

We conducted this study in Albuquerque, NM, USA (35.0844° N, 106.6504° W) and nearby wildlands (34.3417° N, 106.9733° W) located within The Middle Rio Grande Basin (MRG). We received a permit (#19_20R) for this study which was issued by Jon Ertz of the Fish and Wildlife services and accepted by Jennifer Rudgers, director of the Sevilleta LTER. MRG ecosystems are of particular interest because they have evolved under human influence for at least 12,000 years [25]. Disturbances in the MRG ecosystems contribute to soil compaction, lower plant productivity, desertification, invasion of non-native biota, and reduced biological diversity [26]. Albuquerque specifically was the ideal city to study the effects of urbanization because it has recently been expanding at a rapid rate and because it includes a network of desert remnant preserve within the city limits (Albuquerque’s Open Space Network).

We conducted surveys of *Cylindropuntia imbricata* (tree cholla) and its ant visitors across urban, suburban, and wild sites (Seven plots for each site type, n = 21). Plots contained at least 10 tree cholla (210 trees total). Urban sites were all part of the Open Space Network within the city of Albuquerque. These urban open spaces are swaths of land protected from development, but still exposed to mobile urban stresses, such as light and pollution. Suburban sites were in the eastern part of Albuquerque and in Socorro (a town ~122 km south of Albuquerque and ~32 km south the Sevilleta NWR). Wildland sites were located along Route 60 and within the Sevilleta National Wildlife Refuge. We conducted surveys in the summer from July 14^th^ to 28^th^ 2018.

#### Plant survey

We performed surveys of 10 randomly selected tree cholla plants in 30 x 30 m plots (n = 210 tree cholla). When there were more than 10 tree cholla that met survey criteria in a 30x30m area, survey plants were randomly selected by flipping a coin. To be included in the survey, tree cholla plants needed to contain at least one branching segment and their height needed to be under 120cm. To assess variation in plant size, we measured the number of terminal segments, height, and trunk width of each tree cholla.

#### Ant survey

We performed ant surveys from 6:30am-11:30am to maximize the number of ant species who were actively foraging [21]. We then scored the ant activity of three separate branch segments: (i) the highest segment on the plant, (ii) an adjacent segment, and (iii) a segment haphazardly chosen from another region of the plant. We classified the number of ants observed on each segment for one minute and recorded the ant score using a coarse scale (1 = 1–10, 2 = 11–20, 3>20 ants). For both the survey and manipulative experiment below, we assigned morphospecies to each unique ant species and collected voucher specimens with an aspirator after we finished surveying all three segments. We identified all voucher specimens to species using a stereomicroscope and dichotomous key [27].

#### Statistical analyse

We first calculated the coefficient of variation (CV) of height and segment count for each site. The CV shows how much the data varied from the population mean and is calculated by dividing the standard deviation by the mean. We chose to focus on the CV rather the mean because examining variability in response to a disturbance allows us to better predict disturbance-driven change [28]. We then found the mean CV for all surveyed plants in each site type ± 1 standard error of the mean. We used Shapiro-Wilk tests to assess the parametric assumption that residuals were normally distributed and Levene’s test to assess the parametric assumption that variances were homogeneous. These tests revealed that the number of segments violated the assumption that variances among groups are homogeneous, whereas plant height did not violate either assumption. Consequently, to determine if tree cholla size variations were significantly different by site type, we used a one-way ANOVA for height and a Kruskal–Wallis one-way analysis of variance test (KW-ANOVA) for segment count. All statistical analyses were conducted in Origin 2019 (64 bit) v. 9.6.0.172, unless otherwise stated.

We assessed both ant species composition and ant activity levels on *C*. *imbricata* plants. To assess ant activity, we pooled ant scores for each segment, examining ant activity at the plant level. The ant activity data violated assumptions of parametric statistical models (above), so we performed a K-W ANOVA test followed by a Dunn’s post hoc test to statistically evaluate differences in ant activity among site types. Next, we assessed the relative ant composition of the different site types using an ordination (Primer-E v7.0.13). Specifically, we first visualized ant composition using a non-metric multidimensional scaling (NMDS) plot using the presence-absence of ant species at each plant to construct a Bray-Curtis resemblance matrix. We added a dummy variable of 1 to remove any matrix distortions caused by zeros. We used this dissimilarity matrix to construct the NMDS plot with 100 restarts, with a Kruskal fit scheme 1, minimum stress of 0.01. The 2-dimensional stress for the NMDS ordination never exceeded 0.25. We tested for site type (urban, suburban, wild) using a PERMANOVA main test followed by post-hoc pairwise PERMANOVAs to identify which site types significantly varied from one another. To determine which species contributed the most to the dissimilarity in each site type we conducted a SIMPER analysis with a Bray-Curtis similarity measure on an ant species presence-absence matrix. We used a one-way design with site type as the factor and a 90% cut-off percentage (Primer-E v7.0.13). Finally, we conducted a PERMDISP analysis to investigate the homogeneity of multivariate dispersions (Primer-E v7.0.13), one measure of β-diversity.

### Nectar supplement experiment

#### Site selection

We examined the effect of increasing mutualism benefits (i.e. extrafloral nectar) across urban and wild sites in and around Albuquerque. To do this, we conducted a nectar supplement experiment in Albuquerque’s open spaces and in the Sevilleta National Refuge (High = ‘Urban’ and Low = ‘Wild’, Seven sites for each site type n = 14). All sites were ≥200 m away from each other and contained three 10 x 10 m plots ≥50 meters apart. Each of these plots contained two focal plants (control and supplemented) with at least four other non-focal plants in each plot (total of 84 trees). Focal plants needed to meet the same criteria as plants from the surveys. This experiment was conducted in the summer from July 10^th^ to 24^th^ 2019. The month of July typically experiences a moderate amount of rain for desert ecosystems (~40 mm).

#### Nectar supplementation

We supplemented tree cholla with artificial nectaries by hanging three inverted 1.5 mL microcentrifuge tubes ~60 cm apart from one another on each cholla (inversion allowed for capillary action to provide a steady outflow of nectar). The artificial nectaries of supplemented plants contained 1mL of 20% sucrose with 80% water while the artificial nectaries on control plants were empty.

#### Ant aggression trials

To assess ant aggression in response to nectar supplementation, we conducted ant aggression trials the morning following nectar supplementation. To do this, we placed a *Drosophila melanogaster* larva (hereafter, we will refer to these larvae as proxy prey), on two focal segments located near the artificial nectaries. Prior to adding it to the plant, we selected proxy prey that were similar in size and developmental stage (1^st^– 2^nd^ instar proxy prey). We conducted all trials for one site between the hours of 6:30am-11:30am, the approximate period when ants have been found to be most active on tree cholla [20]. Two individual trials with a single proxy prey were conducted for 15 minutes or until proxy prey were removed from the plants (n = 168). Trials were conducted on opposite sides of the cholla and were at least 5 cm from an artificial nectary. Once we placed a proxy prey on a nectary located on a terminal segment (>106 cm above the ground), we recorded the time it took for ants to discover (probe with antenna), attack (bite, spray with formic acid, sting), and remove the proxy prey from the nectary, segment, or plant; the proxy prey was considered removed from the plant if ants had carried proxy prey within 50 cm from the ground by the end of the trial. We assessed removal at the level of nectary, segment, and plant to determine if the effect of supplementation is weakened depending on distance from the source. We collected voucher specimens of all ants present with an aspirator after we finished ant aggression trials for each focal plant and identified them as described for the surveys.

#### Statistical analysis

We first assessed how site type and nectar treatment affected ant composition using the same methods as described for the survey. To assess ant aggression, we first examined the amount of time it took for each behavior to occur as a function of site type and treatment. We used a Kaplan-Meier (K-M) Survival analysis with log-rank χ2 test to examine the amount of time it took ants to discover, attack, or remove prey. This type of analysis is particularly useful when data are right-censored (i.e., the trials ended before some behaviors were observed). We then assessed the proportion of successful behaviors (i.e., number of discoveries, attacks, removals/total trials) per plant factored by site type and nectar treatment. We used randomization tests to evaluate differences among treatments because our residuals were not normally distributed [29]. Specifically, we compared a null distribution of F-values from randomly assigned response variables to the observed F value, assuming significance with an alpha of 0.05. We used randomization test equivalents of ANOVA by encompassing Proc GLM code within a SAS randomization test macro program [30].

## Results

### Survey

#### Plant survey

Overall, tree cholla plant sizes in suburban sites were more variable than those in either of the other two site types; however, these patterns were non-significant for both height and segment count (S1 Fig, Kruskal-Wallis: χ2 = 3.213 *P*_Seg_ = 0.201, ANOVA: F = 2.280 *P*_Ht_ = 0.131).

#### Ant survey

**Fig 1:**
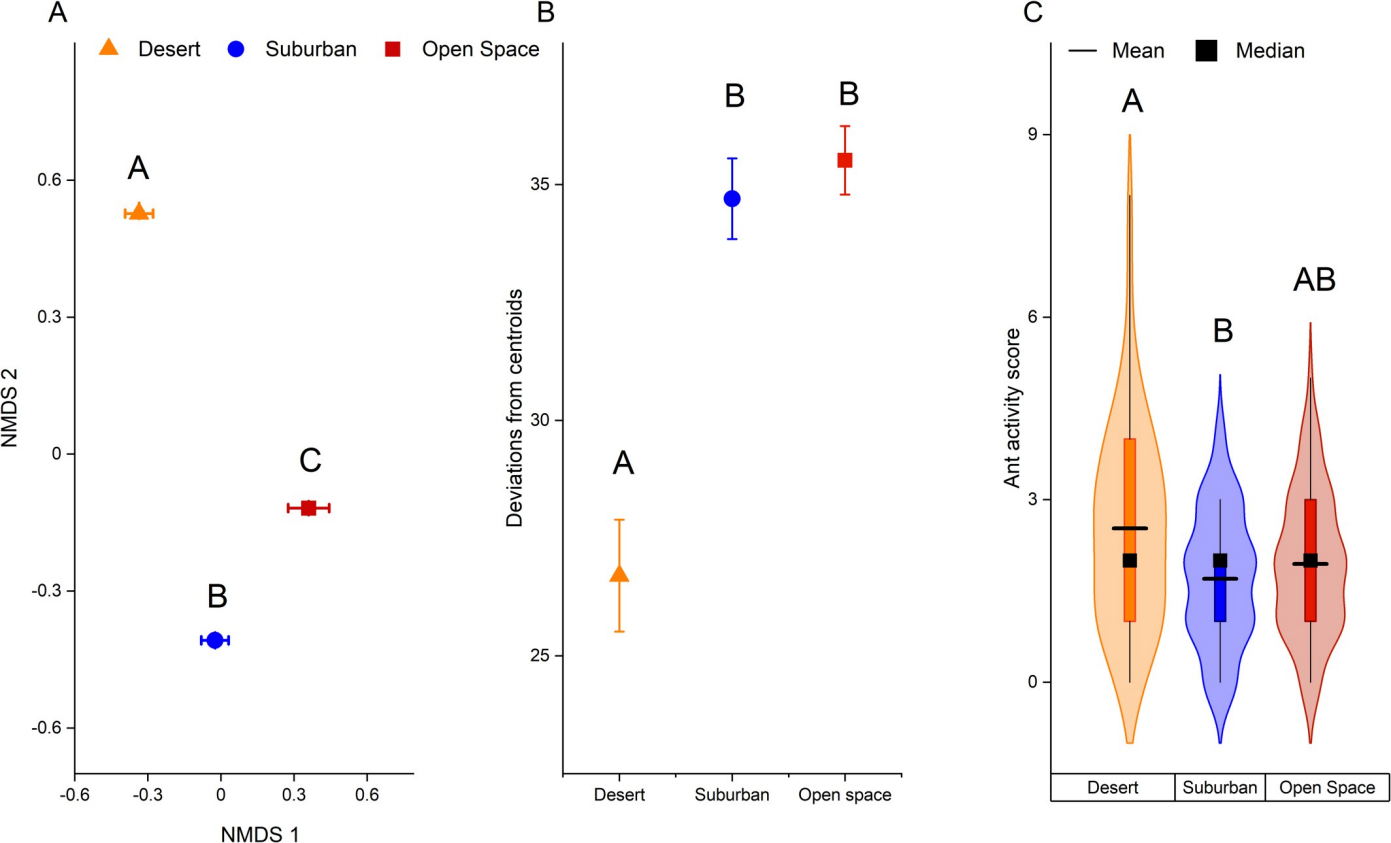
Ant α-diversity, β-diversity, & activity levels across sites with varying levels of urbanization (high = ‘urban’, intermediate = ‘suburban’ and low = ‘wildlands’). (A) Centroids ± 1 SEM from a non-metric multidimensional scaling (NMDS) ordination showing ant species present in response to site type; 2D stress = 0.16, PERMANOVA: *P*_*Site type*_ = 0.0001; different letters denote significant differences (post-hoc pairwise PERMANOVA P<0.05). (B) Mean distance from centroids ± 1 SEM (a measure of β-diversity); Site types had significantly different β-diversity across all sites (Permdisp P_ST_ = 0.0001). Different letters represent significant differences among site types (post-hoc pairwise PERMANOVA: *P*<0.05). (C) Violin plot depicting variation in ant activity across sites with varying levels of urbanization. Ant activity was scored on 3 tree cholla segments (1 = 0–9 ants, 2 = 10–19 ants, 3 = 20–29 ants). Horizontal lines represent means while squares represent medians. Inside the box plot is the interquartile range with the tails being the 95% confidence interval. The shaded region is the density probability distributions, given the variability of each group. There was a significant effect of site type on ant activity scores (Kruskal-Wallis test, *P* = 0.008); site types with different letters were significantly different from one another (post-hoc Dunn test: *P<*0.05).

Across all sites, ant communities were very distinct. We collected a total of 719 ants from 15 genera and 26 species. Of the 210 trees surveyed, only 12 trees had no ants present. Only four species were found in all three site types: *Camponotus vicinus*, *Crematogaster dentinodis*, *Dorymyrmex flavus*, and *Forelius mccooki* (S1 Table, S2 Fig). Ant α-diversity varied significantly by site type, as evidenced by significant shifts in species composition in wildlands, suburbs, and urban sites (Fig 1A, PERMANOVA main test: Pseudo-F = 23.212 *P* = 0.0001). Across the urbanization gradient, wildlands had the most distinct ant fauna (Fig 1A and 1B, pairwise PERMANOVA t_*S-D*_ = 6.0518 *P*_*S-D*_ = 0.0001, PERMANOVA t_*D-O*_ = 4.6999 *P*_*D-O*_ = 0.0001, PERMANOVA t_*S-O*_ = 3.6403 *P*_*S-O*_ = 0.0001).

The top contributor to differences among suburban and wild sites, *Crematogaster navajoa*, was common in wild sites but absent from suburban sites. Similarly, *Cr*. *dentinodis*, which had the second greatest contributions to compositional differences among wild and suburban sites was abundant in wild sites but rare in suburban sites. The other two top contributors to differences among suburban and wild sites, *Camponotus vicinus* and *Tetramorium spinosum*, were abundant in suburban sites but rare in wildland sites (S3A Table). Interestingly, *Ca*. *vicinius* and *T*. *spinosum* also contributed the most to differences among suburban and urban sites, with *T*. *spinosum* being absent from urban and *Ca*. *vicinius* occurring in ~65% fewer plants in urban areas relative to suburban sites. *Forelius mccooki* and *Formica limata* also strongly contributed to differences between suburban and urban sites; both species were much more common in urban sites than they were in suburban sites (S3B Table). Finally, *Cr*. *navajoa*, *Cr*. *dentinodis*, *Forelius mccooki*, and *Formica limata* were the greatest contributors to ant species compositional differences among wild and urban sites. *Crematogaster navajoa* and *Cr*. *dentinodis* were 409% and 571% more common in wildlands than in urban sites. In contrast, *Forelius mccooki* had 42% higher occupancy in urban sites than in wildland and *Formica limata* was absent from wildlands but relatively common in urban sites (S3C Table).

We found similar trends for among-site (β) diversity, with a significant effect of site type (Fig 1B, F_ST_ = 26.615 P_ST_ = 0.0001) driven largely by differences between wildland sites and both other site types (PERMDISP Pairwise t_S-D_ = 5.4622 *P*_S-D =_ 0.0001, t _OS-D_ = 6.3271 *P*_OS-D_ = 0.0001). The β- diversity of ants in urban sites did not significantly vary from that of ants in suburban sites (PERMDISP Pairwise t _S-OS_ = 0.72686 *P*_S-OS =_ 0.4895, Fig 1B).

Site type also had a significant effect on ant activity (Kruskal-Wallis: *P* = 0.008, χ2 = 9.550). Ant activity scores in the wildlands, predominantly *Crematogaster* spp., were significantly higher than activity scores of ants found in suburban sites, predominantly *T*. *spinosum and C*. *vicsnus* (Fig 1C, z = 3.043 Dunn test, *P* = 0.007). There were no significant differences in ant activity between urban and suburban sites (Dunn test, z = -1.053 P = 0.887) and urban and wildland sites (Dunn test, z = 1.990 P = 0.140).

### Nectar supplementation experiment

#### Ant composition

Across all site types, ant communities were very distinct while nectar supplementation did not affect the composition of ants on *C*. *imbricata* plants. We found a total of 14 ant species from 9 genera across all plants in the experiment (S3 Table, S4 Fig). Of the 84 experimental trees, only one tree had no ants present. Ant composition was significantly different among site types but was not significantly influenced by the nectar treatment or the interaction between site type and the nectar treatment (PERMANOVA main test Pseudo-F_ST_ = 16.517, *P*_ST_ = 0.0001, Pseudo-F_TRT_ = 0.47931 *P*_TRT_ = 0.7847, Pseudo-F_INT_ = 0.61941 *P*_INT_ = 0.6833). The species contributing the most to differences between wildlands and urban were *C*. *navajoa* (26.53%), *F*. *pruinosus* (14.81%), *Tetramorium immigrans* (14.06%), and *C*. *dentinodis* (10.16%) (S4A Table). Wildland sites had higher abundances of both *Crematogaster* species, while urban sites had higher abundances of *F*. *pruinosus* and *T*. *immigrans*. Nectar supplementation had no significant effect on ant species composition, with extremely similar levels of occurrence for all species who contributed most to differences (S4B Table).

**Fig 2:**
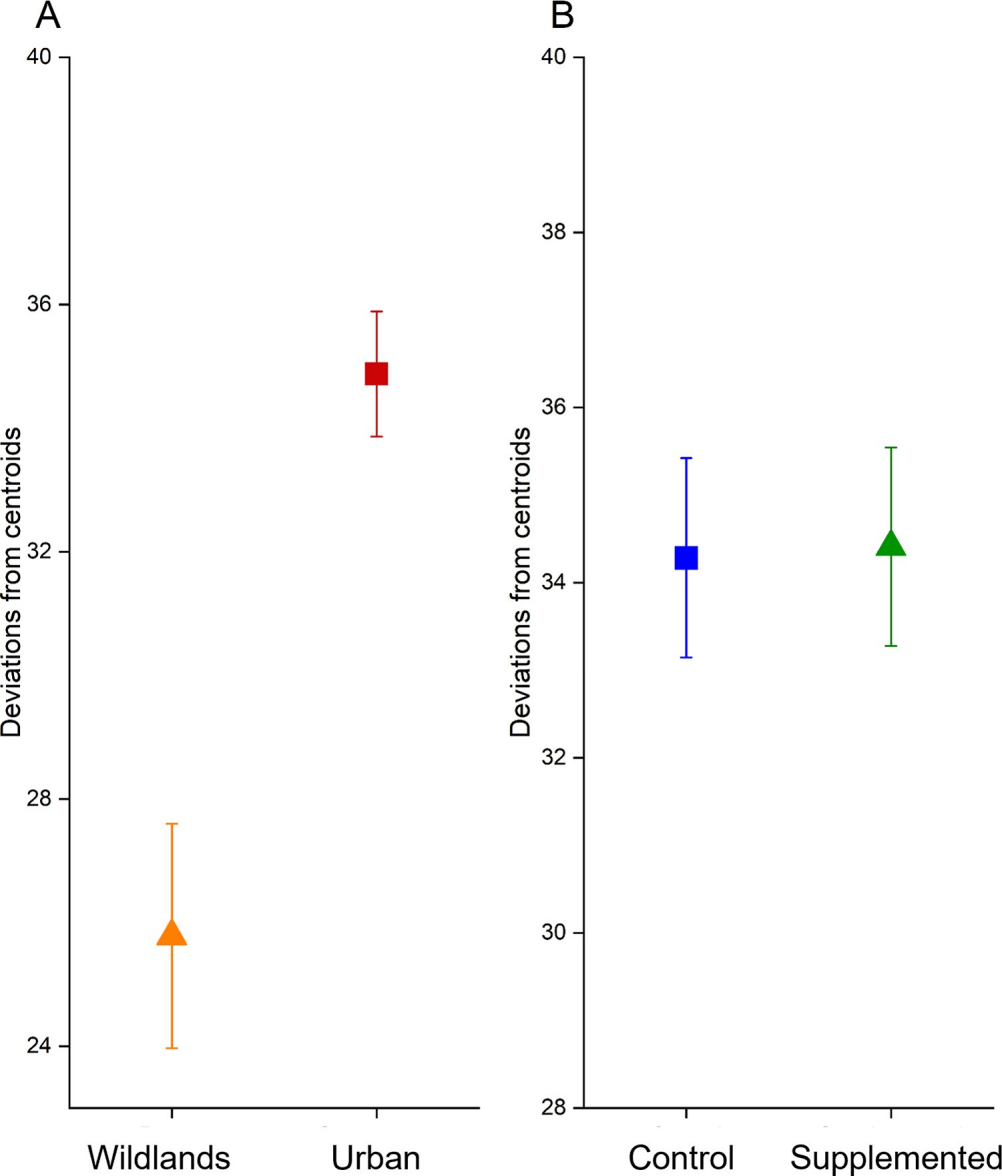
β-diversity (deviations from centroids) across sites with differing levels of (A) urbanization (High = ‘Urban’ and Low = ‘Wildlands’) and (B) nectar supplementation (High = Supplemented and Low = Control). Deviations from centroids were significantly different between site types but not different for nectar supplements *vs*. control plants. (Permdisp P_ST_ = 0.0005, P_TRT_ = 0.9509). Factors with different letters were significantly different according to a post-hoc pairwise Permdisp (P<0.05) Error bars represent ± 1 standard error of the mean.

Similarly, there was a significant effect of site type on β-diversity, with urban sites having higher β-diversity than wildland sites (Fig 2A, F_ST_ = 19.12 P_ST_ = 0.0005). We found no such effect of nectar treatment with supplemented and control plants having nearly identical β-diversity (Fig 2B, PERMDISP, F_TRT_ = 0.0060 P_TRT =_ 0.9509).

#### Ant aggression towards proxy prey

**Fig 3:**
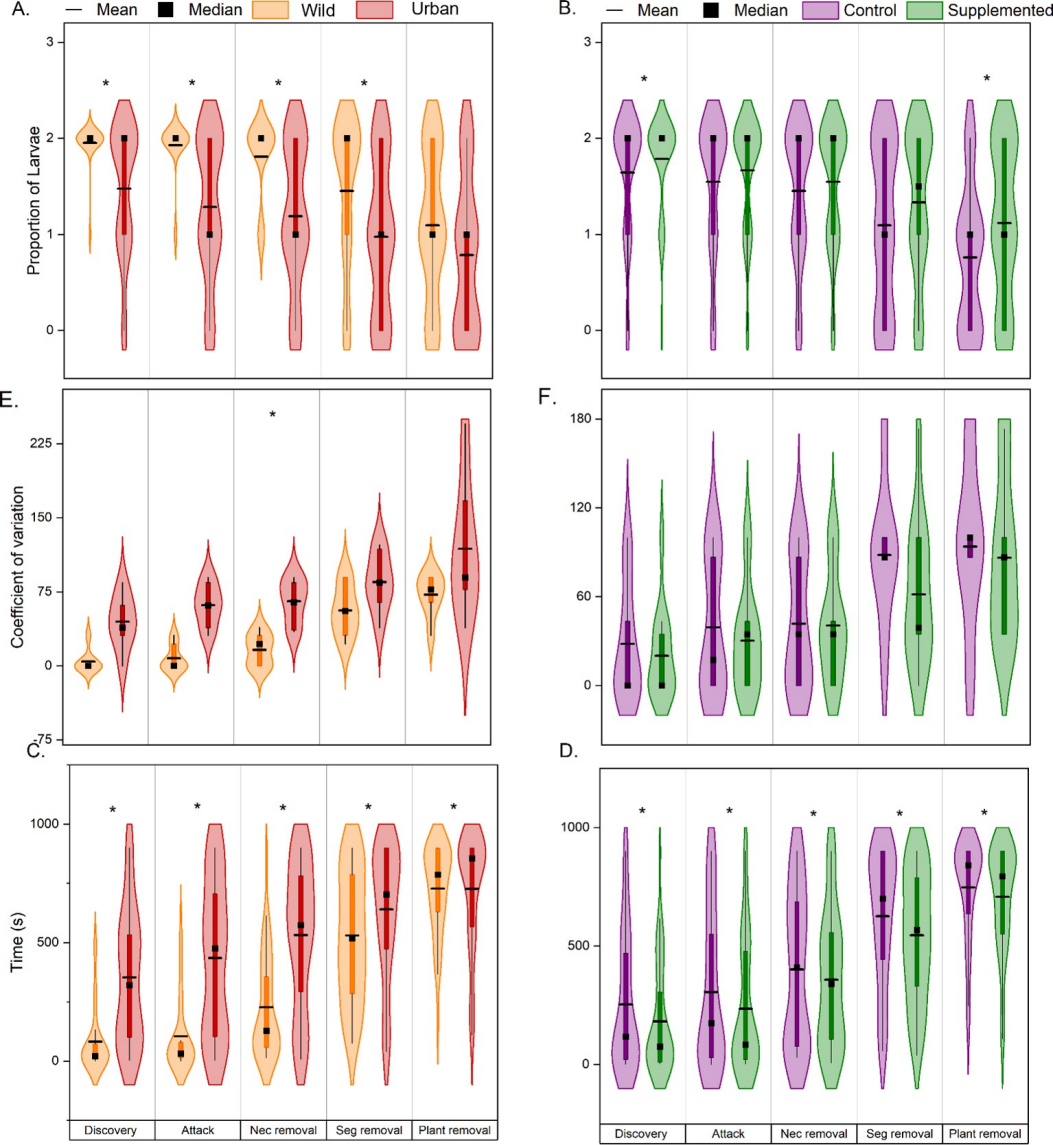
Variation in ant behaviors across sites with different levels of urbanization (left panels) and nectar supplements *vs*. control plants (right panels). (A, B) The number of *D*. *melanogaster* proxy prey that ants discovered, attacked, or removed (from nectaries, segments, and whole plants); (C, D) the relative time it took ants to perform these behaviors; (E, F) the CV for number of proxy prey that were subject to each behavior. Violin plots are as described in Fig 1C. Factors with asterisks were significantly different according to the relative statistical test (P<0.05).

We found that both site type and nectar supplementation significantly influenced most ant behaviors performed on proxy prey, with ants on supplemented plants and in desert sites more likely to perform the behaviors. However, site type had a consistently stronger effect than nectar supplementation. Ants were 24% more likely to discover proxy prey in wildland sites than in urban sites (Fig 3A; Table 1; ANCOVA: F_ST_ = 14.46, P_ST_ = 0.0014) and 8% more likely to discover proxy prey on supplemented plants than on control plants (Fig 3B, Table 1, ANCOVA: F_TRT_ = 6.37, P_TRT_ = 0.0138). Site type and ant species significantly affected the proportion of proxy prey that ants attacked. Ants in wildland sites were ~33% more likely to attack proxy prey than in urban sites (Fig 3A, ANCOVA, F_ST_ = 11.37, P_ST_ = 0.0025). While significant, ant species identity had a lower effect size than that of site type (ANCOVA, F_TRT_ = 1.83, P_AS_ = 0.0284). Site type was the only factor that significantly affected the proportion of proxy prey that ants removed from nectaries, and desert ants were ~34% more likely to remove proxy prey from nectaries (Fig 3A, ANCOVA, F_ST_ = 8.38 P_ST_ = 0.0059). Again, site type was the only factor that significantly affected the proportion of proxy prey that ants removed from segments; ants were 33% more likely to remove proxy prey from segments in desert sites (Fig 3A, ANCOVA: F_ST_ = 4.38, P_ST_ = 0.0378). Unlike previous behaviors, removal from plant rates were only significantly affected by nectar supplementation, with ants being 32% more likely to remove proxy prey on supplemented plants than control plants (Fig 3B, ANCOVA: F_TRT_ = 4.07, P_TRT_ = 0.0496. Site type also had a significant effect on the variability of discovery, attack, and removal from nectaries, with higher levels of variability in urban compared to wildland sites (Fig 3C, Mann–Whitney: U _ST-D_ = 4.5, P_ST-D_ = 0.0076, U _ST-A_ = 0.5, P _ST-A_ = 0.0021, Two-Sample t Test: t_ST-NR_ = -4.681 P_ST-NR_ = 5.32x10^-4^). Variability in removal from segment and plant rates were unaffected by site type in addition to all behaviors of the supplementation treatment (Fig 3C and 3D Mann–Whitney U_TRT-D_ = 106.5, P_TRT-D_ = 0.6824, U_TRT-A_ = 103, P_TRT-A_ = 0.8263, U_TRT-NR_ = 93.0, P_TRT-NR_ = 0.8305, U_TRT-SR_ = 125.5 P_TRT-SR_ = 0.2031, U_TRT-PR_ = 108.5 P_TRT-PR_ = 0.6377, U_ST-PR_ = 12.5, P_ST-PR_ = 0.13199, Two-Sample t Test: t_ST-SR_ = -1.954, P_ST-SR_ = 0.0744).

**Table 1:** Results from ANCOVA of proportional data. We examined both the effect size (F) and the probability our results occurred from random chance (P).

Behavior	Factor	F	*P*
Discovery	***Site type***	14.46	0.0014
	***Treatment***	6.37	0.0138
	Site type * Treatment	2.56	0.1156
	Ant species	1.83	0.0753
Attack	***Site type***	11.37	0.0025
	Treatment	2.02	0.1559
	Site type * Treatment	0.34	0.5664
	***Ant species***	1.99	0.0284
Removal from nectary	***Site type***	8.38	0.0059
	Treatment	0.61	0.4347
	Site type * Treatment	0.27	0.6153
	Ant species	1.61	0.0686
Removal from segment	***Site type***	4.38	0.0378
	Treatment	1.65	0.1971
	Site type * Treatment	0.77	0.3795
	Ant species	1.27	0.1802
Removal from plant	Site type	2.50	0.1222
	***Treatment***	4.07	0.0496
	Site type * Treatment	1.45	0.2311
	Ant species	1.34	0.1313

Significant factors are bolded and italicized.

As with proportions of proxy prey, rates at which ants performed aggressive behaviors were significantly affected by nectar supplementation and site type, with supplemented plants and desert sites having quicker behavioral times. Specifically, discovery was 326% faster (Fig 3E, Table 2, K-M Survival analysis: χ^2^_ST_ = 409.95, P_ST_ <0.0001), attacks occurred 315% faster (Fig 3E, K-M Survival analysis: χ^2^_ST_ = 651.60, P_ST_<0.0001), and removal from nectaries and stems was 134% (K-M Survival analysis: χ2_ST_ = 632.37, P_ST_ = <0.0001) and 21% (K-M Survival analysis: χ^2^_ST_ = 69.66, P_ST_ <0.0001) faster, respectively, in wildlands than in urban sites (Fig 3E). Interestingly, there were no significant differences in removal from whole plants between wild and urban sites (Fig 3E). While less extreme, nectar supplements also significantly affected the speed at which ants performed aggressive behaviors. Specifically, ants discovered proxy prey 39% faster (Fig 3F, K-M Survival analysis: χ2_TRT_ = 32.53, P_TRT_ <0.0001), attacked proxy prey 30% faster (Fig 3F, K-M Survival analysis: χ^2^_TRT_ = 34.37, P_TRT_ <0.0001), and removed proxy prey from nectaries, segments, and whole plants 12% (K-M Survival analysis: χ^2^_TRT_ = 15.91, P_TRT_ <0.0001), 15% (K-M Survival analysis: χ^2^_TRT_ = 88.89, P_TRT_ <0.0001, and 6% (χ^2^_TRT_ = 42.06, P_TRT_ <0.0001) faster, respectively, on plants with nectar supplements than on control plants (Fig 3F).

**Table 2:** Results from K-M survival analysis of the times it took ants to perform behaviors associated with defending *C*. *imbricata* plants.

Behavior	Factor	χ2 (Log rank)	*P*
Discovery	***Site type***	409.95	<0.0001
	***Treatment***	32.53	<0.0001
Attack	***Site type***	651.60	<0.0001
	***Treatment***	34.37	<0.0001
Removal from nectary	***Site type***	632.37	<0.0001
	***Treatment***	15.91	<0.0001
Removal from segment	***Site type***	69.66	<0.0001
	***Treatment***	88.89	<0.0001
Removal from plant	***Site type***	5.53	0.0197
	***Treatment***	42.06	<0.0001

Chi-squared values are based on log-rank estimates Significant factors are bolded and italicized.

## Discussion

In this study, we sought to improve our understanding of how ants and their mutualistic partners, tree cholla cacti (*Cylindropuntia imbricata*), cope with multifaceted urban stress. We found that site type strongly influenced ants—both in terms of their composition and their activity on the plant- though plant traits did not show a similar trend in response to site type. Moreover, we found that ants were more aggressive towards proxy prey in wildlands than they were in urban sites. Experimentally increasing nectar availability increased the likelihood that proxy prey would be discovered and removed from the plant, but did not influence the likelihood of attack, nectary removal, or segment removal. Both nectar treatment and site type influenced the speed at which ants performed these aggressive behaviors, with wildland sites and supplemented plants having quicker times for all behaviors. Nonetheless, our results indicate that the effects of site type are much stronger than the effects of changing nectar levels-the currency of this mutualism.

### Wildlands had the most distinct and active ant community

Ant composition varied strongly across different site types. Wildland sites had the lowest number of species with the most distinct community composition, a significant trend for both the survey and experiment. *Crematogaster spp*. ants were the main drivers of this pattern. Wildland plants were more likely be patrolled by *Cr*. *navajoa* and *Cr*. *dentinodis*. Interestingly, another ant species from the same genus has been previously documented as a key partner in this mutualism (*Cr*. *opuntiae*) [20, 21, 31]. Previous studies also found that *Liometopum apiculatum* was the most effective plant protector in this mutualism, yet we did not find this ant in any of the plants in our study. Changes in ant occupancy as a result of other anthropogenic drivers, such as climate change, may explain the differences in the identity of the dominant ants on *C*. *imbricata* in wild sites in our survey compared to previous studies in this system. Future studies that investigate the interface between urbanization and climate change for the dynamics of facultative mutualisms will help resolve the simultaneous effects of climate change and urbanization for ant occupancy of *C*. *imbricata* plants. Furthermore, experimental manipulations of specific abiotic conditions (such as temperature and/or aridity) could help us understand what aspects of urbanization are most likely to underlie this shift in ant species composition.

We found that ant species not previously reported as partners in this mutualism dominated both urban and suburban sites, although ant composition differed between these two human-dominated landscapes. Overall, suburban sites had the highest number of ant species visiting tree cholla plants in addition to having the most distinct ant composition from wildlands. *Camponotus vicinus* and *Tetramorium spinosum* were the main contributors to this difference found in suburban sites. *Forelius mccooki* and *Formica limata* were the species driving differences between urban sites and the other two site types. *Forelius mccooki* has a high thermal tolerance [21], which allows them to be active when other ants are not. This may allow them to be more active under the elevated temperatures generated by the urban heat island effect. *Formica limata* are reported to show low levels of aggression [27] and thus unlikely to provide a strong defense. Interestingly, these trends align with what has been previously observed, where accumulation of generalist species in urban areas outweighs the loss of native species, ultimately leading to an increase in diversity at the local or regional level [5, 32, 33].

Variation in ant activity across sites can likely be explained by the ant composition found in each site type. Wildland ants were significantly more active than suburban ants, yet not urban ants. One explanation for this increased activity in wildland sites is that our morning survey time effectively captured the activity peaks of specialized desert ants while missing the activity peaks of the generalist species in more urbanized environments (which may have occurred later in the day). Wildland sites were consistently dominated by the morning-active *Crematogaster spp*. (Miller & Savage, unpublished data), which were less common in urban sites and rare at suburban sites. In contrast, Fitzpatrick et al. (2014) recently demonstrated that generalist ant species have higher thermal maxima than more specialized ant species, and that their highest foraging rates occur later in the day, when temperatures are closer to their thermal maxima [21]. We detected primarily generalist ant species in our suburban sites and conducted our surveys before temperatures became very high. Therefore, we hypothesize that ant activity rates may be higher in suburban sites later in the day. However, our study was unable to detect ant activity later in the day because we restricted our surveys to mornings. Future studies across different time periods could elucidate variation in ant activity across sites with varying levels of urbanization at diurnal, crepuscular, and nocturnal times.

### Ant aggression was stronger in wildlands than in urban sites

Wildland ants also exhibited more intense aggression towards proxy prey than those in urban sites. Discovery, attack, removal from nectary, and removal from segment occurred significantly more often and at a quicker rate in wildland sites compared to urban sites. Site type had a different trend with removal from plant, with significant differences in the amount of time it took to perform this task but no effect on number of proxy prey being removed. This higher aggression found in the wildlands was affected by ant identity, although site type had a much stronger effect. This variation in aggressive behaviors may be due to resource availability of respective sites. Since some resources are less available in the wildlands compared to urban areas, where anthropogenic food sources are more common, additional nectar in desert sites may be a more valuable commodity for a diversity of animals. For example, Łopucki et al (2021) compared direct encounters of striped field mice in urban and wildlands and found that wildland mice were significantly less likely tolerate other animals at baited camera traps [34]. This resource imbalance may be exacerbated for ants visiting *C*. *imbricata* in desert ecosystems since both the ants and plants are already nutrient limited. Alternatively, EFN may be more nutrient-rich in wild systems, where plants are not exposed to intense urban stress. Previous research has shown that higher quality nectar is more attractive to ants visiting extrafloral nectaries [35]. Future studies should assess the concentration of sugars and amino acids in *C*. *imbricata* nectar across habitats with varying levels of urban stress to test this hypothesis.

As in the survey, wildland sites had significantly different ant composition than urban sites. These differences were primarily driven by increased abundances of *C*. *navajoa* in wildlands compared to urban sites. In contrast, the generalist ant species *Forelius pruinosus* and *Tetramorium spinosum* were more abundant in urban sites than they were in wildland sites and were ranked the 2^nd^ and 3^rd^ greatest contributors to differences in ant compositions among site types (with similar effects on composition). These results suggest that the genus known to interact in food-for-defense mutualisms, which is common in wildlands, likely provides better defense than the genera that are non-mutualistic partners in wildlands, but are commonly found in urban sites. This difference among sites with varying levels of urban stress aligns with patterns documented for bee pollinators in urban systems, where specialist species convey greater fitness benefits than generalists. Specifically, urbanization has been shown to lead to a decline in bee specialists more than generalists, directly altering plant-pollinator networks [36, 37].

The importance of these kinds of studies is underscored by the differences we found in ant composition between site types. Under or undeveloped urban habitats such as urban open spaces, provide refuges where native species can persist and act as source populations for the most disturbed urban habitats [9]. Even as urban wildlands act as refuges from the most extreme disturbances; they still experience overall pressures of urbanization [33]. Mitigation of these stresses as well as greater habitat connectivity could greatly improve biodiversity conservation in cities, including Albuquerque.

### Effects of EFN subsidies on ant aggression varied but were consistently weaker than site type

Ants on supplemented plants discovered and removed proxy prey from cholla plants quicker and more often than ants on control plants. Attack, removal from nectary, and removal from segment only occurred at quicker rates (not more often) on supplemented plants. Similarly, Flores-Flores et al. (2018) found quicker discovery and attack times on plants with nectar supplements and suggested that these quicker times were due to increased ant recruitment found on supplemented plants [38]. Furthermore, González-Teuber et al. (2012) showed EFN investment positively correlated with efficacy of ant defense and was unaffected by presence of EFN robbers who provided no defense [24]. In contrast, Savage and Whitney (2011) found that the effects of nectar supplementation on ant aggressive behaviors depended on ant identity, with a widespread invasive ant (*Anoplolepis gracilipes*) responding with dramatically increased aggression in response to nectar supplements while co-occurring ants displayed consistent levels of aggression regardless of the amount of nectar on the plants [17]. All of these studies were conducted in different ant-plant food for defense mutualisms, suggesting that response to nectar availability and quality may be dependent on the plant species providing the nectar. Additionally, we only supplemented one aspect of EFN (sucrose), thus we may not have captured the full influence of EFN that includes other components such as amino acids and monosaccharides.

### Tree cholla size did not significantly differ across the urbanization gradient

Despite turnover in the identity of ant guards, which have been shown to influence plant growth (18), tree cholla were statistically similar in all of the size measurements we examined and were equally abundant in the urban and wildland sites (S. Lynch, personal observation). An explanation for this apparent paradox may be that mutualistic partners have different life spans, with tree cholla as a long-lived perennial, being visited by the shorter-lived defender ants. Such a difference in partner life spans leads to greater likelihood that the longer-lived partner will interact with multiple partner species throughout their life span [39]. Alternatively, *Crematogaster spp*. are still present in urban sites, although at much lower abundances, so it is possible that urban tree cholla may still be able to associate with more aggressive ant species at crucial life stages as previously demonstrated in wildland sites [19]. Palmer et al. (2010) found that tradeoffs between survival and reproduction over the lifespan of the longer-lived partner allows for cooperation to persist even while ants’ short-term effects range from cooperative to antagonistic [39]. Similarly, tree cholla may be able to mitigate effects of urban stress on fitness by aligning with more effective ant defenders within urban sites during crucial life stages. Since natural selection acts on lifetime fitness, to accurately encapsulate ant defense on fitness of tree cholla, temporally dependent effects of defense should be further addressed in an urban setting. Additionally, a nectar survey along with ant aggression trials could further elucidate why wildland ants are more aggressive than urban ants. Plants exposed to urban stress may be unable to invest as many resources into nectar production. There may also be fewer herbivorous insects in urban sites than in wildlands. Denys and Schmidt (1998) found that herbivorous insect richness declined as urbanization increased [40]. If a similar trend exists in suburban and urban sites, then this lower herbivore pressure could explain why tree cholla seem unaffected by this relatively poor ant defense. Finally, urban plant responses to other environmental factors that we did not measure, such as soil conditions, water availability, and temperature, could have overwhelmed any effects of ant mutualists in these systems.

We observed a non-significant trend where wildland tree cholla varied the least compared to the other site types. In a similar survey, Hou et al. (2019) examined how reproductive traits, seed set and flower production, varied with urbanization. They found that as urbanization intensified, the number of flowers increased while the seed set ratio decreased. They applied the trade-off hypothesis surmising that reduction in seed-set was a trade-off strategy to be able to produce more flowers, attracting more pollinators [41]. Similar trade-offs in tree cholla traits not measured could explain the lack of difference we observed in tree cholla size. Additionally, life stage may have a significant effect not measured here. For example, tree cholla in reproductive life stages had both increased EFN quantity and quality, while size of tree cholla only correlated with production rates. These differences led to greater benefits for ants foraging on reproductive plants [20]. If true for urban tree cholla, they may be able to align with beneficial ant genera in vulnerable life stages which could explain the lack of difference between size traits between site types.

## Conclusions

In sum, our results demonstrate that ant composition, activity, and aggressive behaviors are strongly divergent across sites with varying levels of urban stress. Furthermore, we showed that behavioral changes associated with urbanization are much stronger than changes associated with variation in extrafloral nectar-the key plant reward in this system. Most urban ecology studies have focused on species diversity measurements, and only recently have studies begun to disentangle how assemblages of interacting species are formed in and affected by urbanization [8]. However, mutualisms are likely to mediate the effects of urbanization on interacting species. The differences that we detected in ant composition suggest that the region in and around Albuquerque, NM (USA) could be becoming simplified in a manner consistent with urbanization-induced biotic homogenization [5]. To completely understand the effects of urbanization on these ant-plant mutualist interactions, all interacting partners need to be studied, including herbivores, pollinators, and other predators. Additionally, future studies that quantify how urbanization affects tree cholla fitness over time will improve our understanding of the consequences of urbanization for this mutualism. Nonetheless, this study provides a foundation and critical information about ant diversity, activity, and behaviors in the face of urbanization.

## Supporting information

S1 Fig Coefficient of variation in plant height and number of segments across sites with differing levels of urbanization (high = ‘urban’, intermediate = ‘suburban’ and low = ‘wildland’). Both size measurements showed no significant effect of site type on plant size (Kruskal-Wallis: P_Seg_ = 0. 0.201, ANOVA: P_Ht_ = 0.131). The line represents the mean while the square represents the median. Error bars represent ± 1 standard error of the mean.
(TIF)

S2 Fig Pie charts of the ant species present across an urbanization gradient in the survey. (TIF)

S3 Fig Pie charts of the ant species present during the supplementation experiment. (TIF)

S4 Fig (TIF)

S1 Table The presence-absence of ant species found across sites with differing levels of urbanization (high = ‘urban open space’, intermediate = ‘suburban’ and low = ‘desert wildlands’). An ‘X’ indicatess presence while an empty space represents absence.
(DOCX)

S2 Table The presence-absence of ant species found across sites with two different levels of urbanization (high = ‘urban’ and low = ‘wildlands’) and across plants with two different levels of nectar supplementation (none = ‘control’ and 3mL 20% sucrose = ‘supplemented’). An ‘X’ indicates presence while an empty space represents absence.
(DOCX)

S3 Table Results from SIMPER analysis showing the top four ant species that contributed to compositional differences between (A) Suburban and desert wildlands, (B) Suburban and urban open space, and (C) Desert wildlands and urban open space.
(DOCX)

S4 Table Results from SIMPER analysis showing the top four ant species that contributed to compositional differences between (A) Urban open space and desert wildlands, (B) Control and supplemented.
(DOCX)
