# Supplementary figures and images for: The changing dynamics of ant-tree cholla mutualisms along a desert urbanization gradient
Source: PLoS One. 2023 Mar 31;18(3):e0280130. doi: 10.1371/journal.pone.0280130 (PMC10065256; doi:10.1371/journal.pone.0280130)

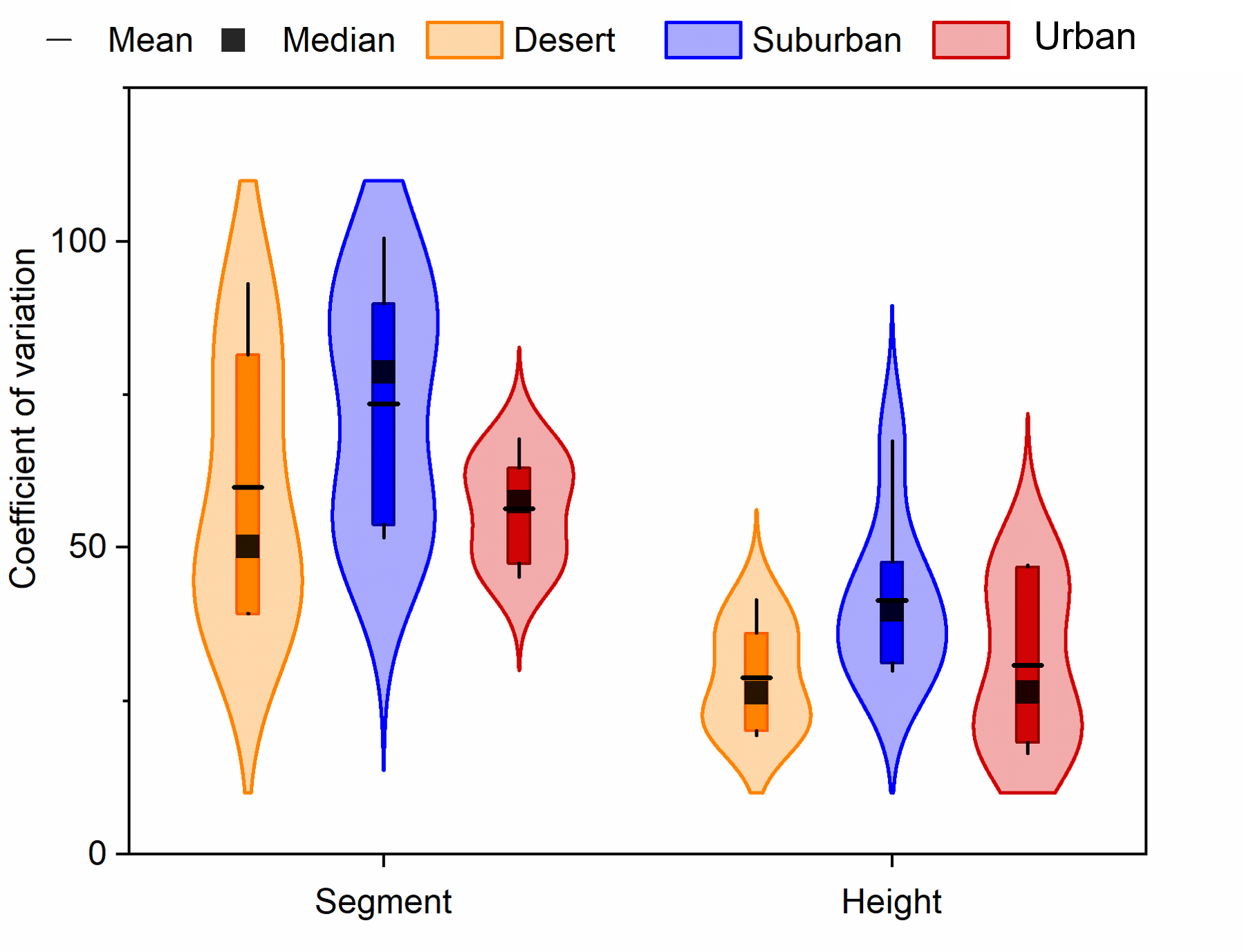

Supplement: S1 Fig — Both size measurements showed no significant effect of site type on plant size (Kruskal-Wallis: PSeg = 0. 0.201, ANOVA: PHt = 0.131). The line represents the mean while the square represents the median. Error bars represent ± 1 standard error of the mean. (TIF) [file pone.0280130.s001.tif]

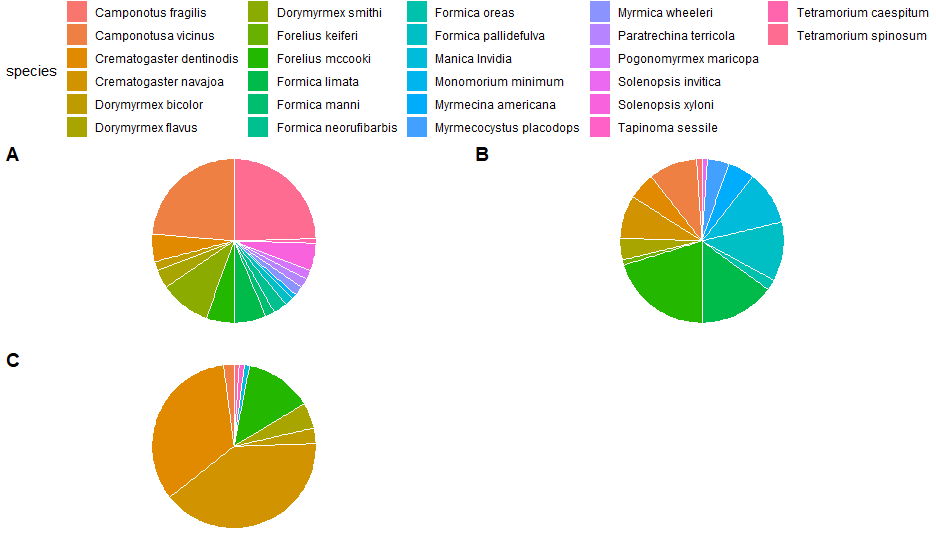

Supplement: S2 Fig — (TIF) [file pone.0280130.s002.tif]

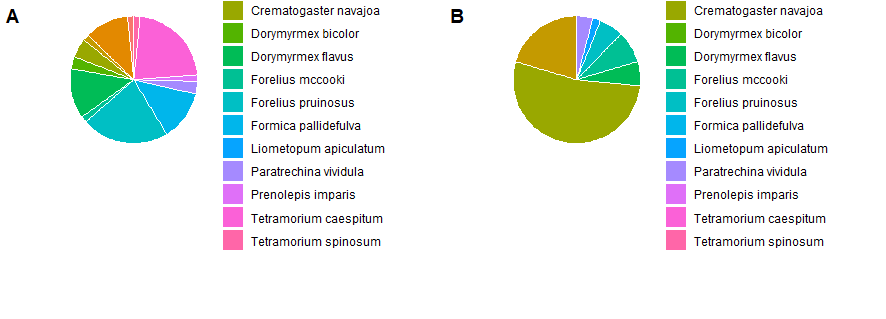

Supplement: S3 Fig — (TIF) [file pone.0280130.s003.tif]

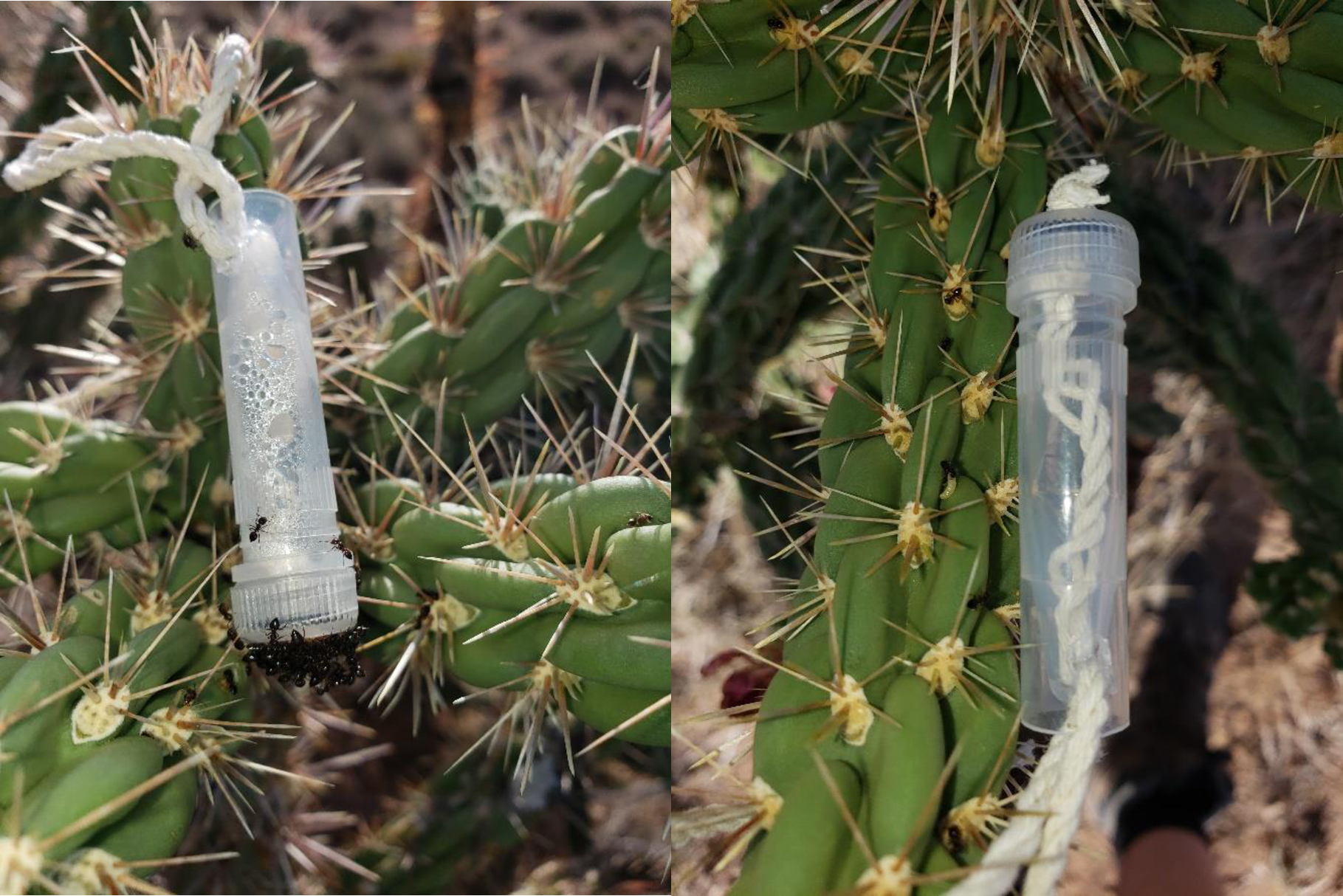

Supplement: S4 Fig — (TIF) [file pone.0280130.s004.tif]
