# Supplementary material for: The changing dynamics of ant-tree cholla mutualisms along a desert urbanization gradient
Source: PLoS One. 2023 Mar 31;18(3):e0280130. doi: 10.1371/journal.pone.0280130 (PMC10065256; doi:10.1371/journal.pone.0280130)
Supplement: S1 Table — An ‘X’ indicatess presence while an empty space represents absence. (DOCX) [file pone.0280130.s005.docx]

**S3 Table 1:** The presence-absence of ant species found across sites with differing levels of urbanization (High=‘Urban’, Intermediate=‘Suburban’ and Low= ‘Wildlands’). An ‘X’ indicates presence while an empty space represents absence.

| **Ant Species** | **Wildlands** | **Suburban** | **Urban** |
| --- | --- | --- | --- |
| *Camponotus fragilis* |  |  | X |
| *Camponotus vicinus* | X | X | X |
| *Fatogaster dentinodis* | X | X | X |
| *Crematogaster navajoa* | X |  | X |
| *Dorymyrmex bicolor* | X | X |  |
| *Dorymyrmex flavus* | X | X | X |
| *Dorymyrmex smithi* |  | X |  |
| *Forelius keiferi* |  |  | X |
| *Forelius mccooki* | X | X | X |
| *Formica limata* |  | X | X |
| *Formica manni* |  | X |  |
| *Formica neorufibarbis* |  | X |  |
| *Formica oreas* |  |  | X |
| *Formica pallidefulva* |  | X | X |
| *Manica invidia* | X |  | X |
| *Monomorium minimum* |  | X |  |
| *Myrmecina americana* |  |  | X |
| *Myrmecocystus placodops* |  |  | X |
| *Myrmica wheeleri* |  | X |  |
| *Paratrechina terricola* |  | X |  |
| *Pogonomyrmex maricopa* |  | X |  |
| *Solenopsis invitica* |  |  | X |
| *Solenopsis xyloni* |  | X |  |
| *Tapinoma sessile* | X |  |  |
| *Tetramorium immigrans* |  | X |  |
| *Tetramorium spinosum* | X | X |  |
