# Supplementary material for: The changing dynamics of ant-tree cholla mutualisms along a desert urbanization gradient
Source: PLoS One. 2023 Mar 31;18(3):e0280130. doi: 10.1371/journal.pone.0280130 (PMC10065256; doi:10.1371/journal.pone.0280130)
Supplement: S2 Table — An ‘X’ indicates presence while an empty space represents absence. (DOCX) [file pone.0280130.s006.docx]

**S4 Table 2:** The presence-absence of ant species found across sites with two different levels of urbanization (High=‘Urban’ and Low= ‘Wildlands’) and across plants with two different levels of nectar supplementation (None=‘Control’ and 3mL 20% sucrose = ‘Supplemented’). An ‘X’ indicates presence while an empty space represents absence.

| Ant Species | Site Type | | Nectar Supplementation | |
| --- | --- | --- | --- | --- |
|  | Wildlands | Urban | Control | Supplemented |
| *Camponotus fragilis* |  | X | X |  |
| *Camponotus vicinus* |  | X | X | X |
| *Crematogaster dentinodis* | X | X | X | X |
| *Crematogaster navajoa* | X | X | X | X |
| *Dorymyrmex bicolor* |  | X | X | X |
| *Dorymyrmex flavus* | X | X | X | X |
| *Forelius mccooki* | X | X | X | X |
| *Forelius pruinosus* | X | X | X | X |
| *Formica pallidefulva* |  | X | X | X |
| *Liometopum apiculatum* | X |  |  | X |
| *Nylanderia vivdula* | X | X | X | X |
| *Prenolepis imparis* |  | X | X |  |
| *Tetramorium immigrans* |  | X | X |  |
| *Tetramorium spinosum* |  | X | X | X |
