# Supplementary material for: The changing dynamics of ant-tree cholla mutualisms along a desert urbanization gradient
Source: PLoS One. 2023 Mar 31;18(3):e0280130. doi: 10.1371/journal.pone.0280130 (PMC10065256; doi:10.1371/journal.pone.0280130)
Supplement: S3 Table — Results from SIMPER analysis showing the top four ant species that contributed to compositional differences between (A) Suburban and desert wildlands, (B) Suburban and urban open space, and (C) Desert wildlands and urban open space. (DOCX) [file pone.0280130.s007.docx]

**S6 Table 4:** Results from SIMPER analysis showing the top four ant species that contributed to compositional differences between (A) Urban open space and Desert wildlands, (B) Control and Supplemented

| (A) Comparison: Site type, Desert wildlands and Urban open Space (P=0.0001) | | | | | |
| --- | --- | --- | --- | --- | --- |
| Species | Occupancy in Open space sites | Occupancy in Desert sites | % Contribution to differences | | Cumulative % |
| *Crematogaster navajoa*  *Forelius pruinosus*  *Tetramorium immigrans*  *Crematogaster dentinodis* | 0.07  0.33  0.33  0.02 | 0.62  0.07  0.00  0.39 | 26.53%  14.81%  14.06%  10.16% | | 26.53%  41.34%  55.40%  65.56% |
| (B) Comparison: Nectar, Control and Supplemented (P=0.7847) | | | | | |
| Species | Occupancy of Supplemented plants | Occupancy of Control plants | | % Contribution to differences | Cumulative % |
| *Crematogaster navajoa*  *Forelius pruinosus*  *Crematogaster dentinodis*  *Tetramorium immigrans* | 0.36  0.24  0.12  0.14 | 0.33  0.17  0.14  0.19 | | 19.59%  16.32%  12.55%  11.54% | 19.59%  35.91%  48.46%  60.00% |
