# Supplementary material for: The changing dynamics of ant-tree cholla mutualisms along a desert urbanization gradient
Source: PLoS One. 2023 Mar 31;18(3):e0280130. doi: 10.1371/journal.pone.0280130 (PMC10065256; doi:10.1371/journal.pone.0280130)
Supplement: S4 Table — Results from SIMPER analysis showing the top four ant species that contributed to compositional differences between (A) Urban open space and desert wildlands, (B) Control and supplemented. (DOCX) [file pone.0280130.s008.docx]

**S5 Table 3:** Results from SIMPER analysis showing the top four ant species that contributed to compositional differences between (A) Suburban and Desert wildlands, (B) Suburban and Urban open space, and (C) Desert wildlands and Urban open space.

| (A) Comparison: Suburban and Desert wildlands (P=0.0001) | | | | |
| --- | --- | --- | --- | --- |
| Species | Occupancy in Suburban plots | Occupancy in Desert plots | % Contribution to differences | Cumulative % |
| *Crematogaster navajoa*  *Crematogaster dentinodis*  *Camponotus vicinus*  *Tetramorium spinosum* | 0.00  0.09  0.37  0.39 | 0.56  0.47  0.03  0.01 | 21.13%  18.78%  14.96%  12.44% | 21.13%  39.90%  54.86%  67.30% |
| (B) Comparison: Suburban and Open space (P=0.0001) | | | | |
| Species | Occupancy in Suburban plots | Occupancy in Open space plots | % Contribution to differences | Cumulative % |
| *Camponotus vicinus*  *Tetramorium spinosum*  *Forelius mccooki*  *Formica limata* | 0.37  0.39  0.09  0.10 | 0.13  0.00  0.27  0.20 | 16.48%  12.60%  11.95%  11.33% | 16.48%  29.08%  41.02%  52.36% |
| (C) Comparison: Desert wildlands and Open Space (P=0.0001) | | | | |
| Species | Occupancy in Desert plots | Occupancy in Open space plots | % Contribution to differences | Cumulative % |
| *Crematogaster navajoa*  *Crematogaster dentinodis*  *Forelius mccooki*  *Formica limata* | 0.56  0.47  0.19  0.00 | 0.11  0.07  0.27  0.20 | 22.93%  20.45%  14.78%  8.77% | 23.93%  43.38%  58.16%  66.93% |
